# From sebocytes to skin engineering human sebaceous organoids within a reconstructed dermal matrix

**DOI:** 10.3389/ftox.2026.1874806

**Published:** 2026-07-31

**Authors:** Tommaso Pusceddu, Luna Ardondi, Maria Pia Cavaleri, Lucia Sileo, Ilaria Vitali, Camila Quezada Meza, Michele Massironi, Marco Massironi, Barbara Zavan

**Affiliations:** 1 Department of Medical Sciences, University of Ferrara, Ferrara, Italy; 2 Symrise Srl, Padova, Italy

**Keywords:** *in vitro* model, lipidogenesis, organoid, sebaceous gland, sebogenesis

## Abstract

**Introduction:**

Sebaceous gland biology depends on the integration of lipid metabolism, tissue architecture, and stromal interactions, yet most available *in vitro* systems fail to capture these features simultaneously. This work reports the generation of a functional three-dimensional (3D) sebaceous gland organoid embedded within a matrix and designed to reproduce the structural and functional properties of native sebaceous tissue.

**Methods:**

Using SZ95 sebocytes, we first established baseline responses in monolayer conditions using linoleic acid (LA) stimulation and pharmacological inhibition with retinol (RTN) and capsaicin (CPS). We then reconstructed a 3D organoid model in which a sebocyte-rich lipidogenic compartment was spatially organized within a matrix-supported microenvironment and associated with an outer stromal-like component. The model was evaluated using morphological, ultrastructural, and immunofluorescence analyses. To further define the mode of action of sebogenic activation in 3D, we analyzed genes related to extracellular matrix remodeling and tissue adaptation.

**Results:**

In both monolayer and 3D models, LA successfully induced a sebogenic program, characterized by increased lipid accumulation and upregulation of PPARγ and FABP4, which was effectively attenuated by RTN and CPS. The 3D model successfully maintained essential compartmentalization, epithelial identity, and stromal organization. Furthermore, transcriptional analysis revealed that LA stimulation in 3D induced coordinated changes in extracellular matrix and basement membrane genes (including MMP13, MMP16, and THBS2), indicating that sebogenesis is directly coupled with structural remodeling and microenvironmental adaptation.

**Conclusion:**

Altogether, these results demonstrate that we successfully reconstructed a functional *in vitro* model of a sebaceous gland organoid and established this platform as a relevant tool for mechanistic studies and for cosmetic screening of compounds targeting sebum regulation and sebaceous gland dysfunction.

## 1 Introduction

The human sebaceous gland (SG) is an integral component of the skin’s pilosebaceous unit, responsible for the production of sebum, a lipid-rich substance that contributes to multiple cutaneous functions, including waterproofing, antimicrobial defense, immune modulation, and the maintenance of epidermal homeostasis (Pappas, 2009; Lovászi et al., 2017; Zouboulis et al., 2022; Mosca et al., 2025). Sebum composition, primarily consisting of triglycerides, wax esters, squalene, and free fatty acids, is finely regulated by hormonal, genetic, and environmental signals (Clayton et al., 2019). Despite its fundamental role in skin physiology and pathology, the sebaceous gland remains one of the least understood appendages of the skin, largely due to the lack of reliable experimental models that mimic its complex structure and functional dynamics *in vitro*.

Sebocytes, the lipid-producing epithelial cells of the SG, undergo a unique differentiation process culminating in holocrine secretion—where mature sebocytes disintegrate to release their contents into the sebaceous duct. This process is tightly coordinated with signals from surrounding keratinocytes, fibroblasts, immune cells, and components of the extracellular matrix. However, most conventional *in vitro* models rely on immortalized sebocyte lines or monolayer cultures that fail to recapitulate the gland’s native 3D microarchitecture or its interactions with adjacent dermal and epidermal compartments (De Bengy et al., 2019; Hou et al., 2022; Tang et al., 2023; Liu et al., 2024).

The limitations of 2D culture systems are particularly evident when attempting to model conditions such as acne vulgaris, seborrheic dermatitis, rosacea, or age-related sebaceous gland atrophy, where spatial organization, paracrine communication, and mechanical context are critical for disease development and response to treatment. While efforts have been made to isolate and expand primary human sebocytes, these cells rapidly lose their differentiation potential and lipid-secreting capacity outside their native environment. Moreover, the absence of mesenchymal support and matrix cues further compromises their functionality and viability over time (Bakhshandeh et al., 2017; Dawson et al., 2019; He et al., 2020; Nilforoushzadeh et al., 2022).

Recent advances in skin tissue engineering have led to the development of full-thickness skin equivalents incorporating both epidermal and dermal components. These models have been instrumental in studying keratinocyte differentiation, barrier formation, wound healing, and inflammation. However, the inclusion of skin appendages—such as sebaceous glands, hair follicles, and sweat glands—within these systems remains a major challenge (Chaudhari et al., 2016; Derman et al., 2025; Dutra Alves et al., 2025; Yadav and Nguyen, 2025). Most available reconstructed human skin models do not contain functional sebaceous glands, and therefore cannot be used to investigate lipid secretion, sebocytes differentiation, or appendage-specific responses to pharmacological agents.

In parallel, organoid technology has emerged as a promising approach for modeling epithelial tissues *in vitro*, offering new opportunities to recapitulate the self-organizing capacity and functional specialization of human organs (Clevers, 2016; Corrò et al., 2020; Hong et al., 2023; Kim et al., 2023). Sebaceous gland organoids (SGOs), derived from progenitor cells or adult sebocytes, have been generated in floating or matrix-supported cultures and shown to mimic some aspects of SG morphogenesis and lipid production. Nevertheless, these organoids are typically cultured in isolation, lacking the structural and cellular complexity of full skin. In particular, they fail to integrate with dermal components such as fibroblasts and ECM proteins, which are essential for glandular homeostasis and signal integration (Sun et al., 2021; Hosseini et al., 2022; Hong et al., 2023; Wang et al., 2026).

The absence of an integrated skin model incorporating SGOs within a dermal context represents a critical gap in dermatological research. Without a physiologically relevant environment that includes fibroblast-derived signals, mechanical support, and epidermal-dermal crosstalk, SG organoids remain incomplete and their applicability for mechanistic studies or therapeutic screening is limited (Filaire et al., 2022; Lee et al., 2024; Wang et al., 2024). Furthermore, such integrated models are necessary to investigate how sebaceous gland function is modulated by aging, hormonal changes, environmental insults, and topical formulations, including cosmetics and dermatological treatments aimed at modulating sebum production. To address this need, the reconstruction of sebaceous gland organoids within a supportive 3D dermal niche offers a compelling strategy (Shafiee et al., 2024). This approach allows for the embedding of SGOs into a biologically active dermal matrix populated with human fibroblasts, mimicking the anatomical positioning and stromal interactions of native sebaceous glands. Co-culturing these structures with keratinocytes on the surface further facilitates the establishment of a layered epidermal component, enabling epithelial-mesenchymal crosstalk and providing a more complete skin architecture.

Such a model would allow the study of SG biology in a controlled, yet physiologically relevant, *in vitro* setting. By reconstructing the sebaceous gland within a dermal context, it becomes possible to dissect the molecular and cellular mechanisms that govern sebocytes differentiation, lipid biosynthesis, and SG-environment interactions. This is particularly relevant for studying diseases characterized by dysregulated sebum production or sebaceous gland hyperplasia, as well as for evaluating the efficacy and safety of active compounds targeting these pathways.

Moreover, this model holds promise as a platform for investigating skin aging, in which sebaceous gland function and structure are known to decline, contributing to xerosis, reduced skin elasticity, and impaired barrier repair. In this context, we present a strategy for engineering sebaceous gland organoids embedded within a reconstructed human dermis, aimed at re-establishing a functional pilosebaceous-like unit *in vitro*. This system enables the spatial and biochemical organization necessary for the development of a physiologically competent SG model, advancing the field of skin modeling and opening new avenues for research in dermatology, regenerative medicine, and cosmetic science.

## 2 Materials and methods

### 2.1 Cell culture

SZ95 cells were cultured in T75 flasks until reaching 70% confluence in a 1:1 DMEM/F12 (Sial Group, Rome, Italy) medium supplemented with 2% fetal bovine serum (FBS, Euroclone, Milan Italy), 1% Penicillin/Streptomycin (P/S, Sial Group), 2 mM L-Glutamine (Sial Group), and 5 ng/mL hEGF (human Epidermal Grow Factor, EuroClone). For lipids assays cells were seeded in 96-well or 24-well culture plates at a concentration respectively of 1.5 × 10^4^ and 5 × 10^4^ cells per well. For gene expression analysis cells were seeded in a 6-well culture plate at a concentration of 2 × 10^5^ cells per well. human dermal fibroblasts (hDF) obtained from ATCC (American Type Culture Collection) were cultured in Dulbecco’s Modified Eagle’s Medium (DMEM, EuroClone, Milan, Italy), supplemented with 1% P/S and 10% FBS. Cultures were maintained at 37 °C in a humidified atmosphere with 5% CO_2_.

### 2.2 3D cell culture

Three-dimensional cultures were established using Vitrogel Organoid-1 (The Well Bioscience) via the Cell Droplet method, following the manufacturer’s protocol. Briefly, SZ95 sebocytes were detached using 0.25% trypsin-EDTA, washed, and resuspended in complete culture medium supplemented with hEGF. A cell suspension of 100 µL containing 3 × 10^5^ cells (3 × 10^6^ cells/mL) was mixed with 200 µL of Vitrogel Organoid-1 and incubated for 15 min at room temperature.

3D cultures were formed by dispensing 20 µL droplets (up to 5 droplets per well) into a 6-well plate pre-filled with complete medium. Plates were incubated at 37 °C in a 5% CO_2_ humidified atmosphere for 15 days, with medium changes every 48 h. Organoid size and structural consistency were monitored via brightfield microscopy. For 3D co-cultures of SZ95 sebocytes and human dermal fibroblasts (hDF), a mixed cell suspension was prepared prior to gel embedding, maintaining an SZ95 concentration of 3 × 10^6^ cells/mL and an hDF seeding density of 5 × 10^5^ cells/mL to promote compartmentalization.

### 2.3 Treatments

To modulate lipid accumulation, monolayer and 3D cultures were exposed to linoleic acid (LA) to induce sebogenesis, or to retinol (RTN) and capsaicin (CPS) as inhibitory agents. Reagents were dissolved in DMSO. The treatment scheme consisted of an initial 24-h stimulation with 100 µM LA (or arachidonic acid). To evaluate the inhibitory effects, LA-stimulated cultures were subsequently treated with RTN (1 µM or 10 µM) or CPS (50 µM) for an additional 24 h. Control samples were treated with vehicle alone (DMSO, final concentration 0.1% v/v) and were time-matched to ensure equivalent total incubation periods across all experimental groups.

### 2.4 Lipid quantification

SZ95 sebocytes monolayer and 3D cultures lipid content was assessed after treatment through Oil Red O (ORO) assay. At the end of the treatment period, the cultures were washed with 1x PBS and fixed by incubation with 4% paraformaldehyde (PFA, Merck, United States) in PBS at pH 7.4, for 15 min for monolayer cultures and 60 min for 3D cultures. The cultures were then washed twice with 1x PBS for 5 min and incubated with a fresh 0.3% Oil Red O solution in 60% isopropanol for 15 min for monolayer cultures and 30 min for three-dimensional cultures. Oil red O 0.3% working solution was obtained by diluting 3:2 a 0.22 µm filtered 0.5% stock solution of Oil Red O powder solubilized in 100% isopropanol. After incubation with the dye, the cultures were washed with 1x PBS until excess dye was removed. The retained dye was then extracted using 200 µL of absolute isopropanol. The resulting extraction solutions were transferred to 96-well plates, and absorbance at 490 nm was measured using a Victor3 spectrophotometer (Perkin Elmer). ORO assay was performed on three independent biological replicates, and statistical analysis was conducted using one-way ANOVA.

### 2.5 RNA isolation and RT-qPCR

For gene expression analyses, SZ95 sebocytes monolayer cultures were seeded in 6-well culture plates at a concentration of 1.5 × 10^5^ cells/well and maintained in culture 24 h prior to treatment. After a 48-h treatment period, the cells were first washed with PBS and then lysed with *TRIzol RNA Isolation Reagents* (Thermo Fisher Scientific) following the manufacturer’s protocol. Briefly, 500 µL of reagent was added to each culture well, and the obtained lysate was transferred to a 2 mL centrifuge tube, to which 100 µL of chloroform was added. The tubes were then vortexed and incubated for 3 min at RT, and then centrifuged at 12′000 x g for 15 min at 4 °C. The upper aqueous phase that contains the RNA was collected from each tube and transferred into a new tube. The total RNA was purified by adding 250 µL of isopropanol to the aqueous phase. The tubes were centrifuged at 12′000 x g for 10 min at 4 °C, and the supernatant was discarded. The RNA pellet was washed twice with 75% ethanol and then centrifuged at 7,500 *g* for 5 min at 4 °C. The pellet was allowed to dry for 10 min, and the total RNA was then resuspended in 30 µL of DNase/RNase-free water. The RNA samples were quantified using a *NanoDrop 2000* (Thermo Fisher Scientific) and then stored at −80 °C.

RNA samples were retrotranscribed with *SensiFAST cDNA Synthesis Kit* (Meridian Bioscience, United States) following the manufacturer’s protocol. For each reaction 100 ng of RNA were used, samples were first diluted to 15 µL with DNase/RNase-free water (Qiagen). To each RNA sample, 4ul of *5xTransAmp Buffer* and 1 µL of *Reverse Transcriptase* - given with the kit - were added. Reaction tubes were then placed into a *LifePro Thermal Cycler* (TC-96, Bioer, Hangzhou, Cina) set with a one-cycle ramp at 25 °C for 10 min, 42 °C for 15 min and 85 °C for 5 min, followed by a cooling ramp to 4 °C. cDNA samples were then stored at −20 °C until use. For the qPCR reaction *SensiFAST SYBR No-ROX Kit* (Meridian Bioscience) was used, following the manufacturer’s protocol. For each gene of interest analysis, a custom set of primers was used (Invitrogen corporation, United States; primers sequences are listed in Table 1). Reaction mixtures were prepared by adding 7.5 µL for *2x SensiFAST SYBR No-ROX mix*, 0.6 µL of each 10 µM primer to a final concentration of 400nM, 4.8ul DNase/RNase-free water (Qiagen) and 1.5 µL of 1:2 cDNA sample to a total reaction volume of 20 µL. Each sample was analyzed in triplicate, and qPCR reactions were performed into a *Rotor-Gene Q 5-plex* (Qiagen) set with a 95 °C activation step for 2 min followed by a 40 cycle 3-step cycling at 95 °C for 5 s, 69 °C for 10 s and 72 °C for 20 s.

**TABLE 1:** Primer set utilized for qPCR analysis.

Primer target	Forward sequence	Reverse sequence
*PPARG*	CAG​GAG​ATC​ACA​GAG​TAT​GCC​AA	TCC​CTT​GTC​ATG​AAG​CCT​TGG
*FABP4*	TGA​CCT​GGA​CTG​AAG​TTC​GC	AAG​CAC​AAT​GAA​TAC​ATC​ATT​ACA​TCA​CC
*CEBPA*	GGA​CTT​GGT​GCG​TCT​AAG​ATG​AG	GCA​TTG​GAG​CGG​TGA​GTT​TG
*B2M*	CTG​GTC​TTT​CTA​TCT​CTT​GTA​CTA​CAC​TG	CAA​ACC​TCC​ATG​ATG​CTG​CTT​A

For ECM related gene analysis, *Rotor-Disc 100 RT* ^*2*^ *Profiler PCR Arrays* (QIAGEN) were used, following the producer protocol. Briefly, master mix was prepared in ice by combining 1,150 µL of *2x RT* ^*2*^ *SYBR Green Mastermix*, 102 µL of cDNA template, and 1,048 µL of RNase-free water. Array wells were then loaded by dispensing 20 µL of the prepared master mix avoiding air bubbles formation. Discs were then sealed by applying the given sealing film through the *Rotor-Disc Heat Sealer*, secured with the locking ring and inserted into the *Rotor-Gene Q rotor* thermal cycler. The following thermal cycling program was set: an initial activation step at 95 °C for 10 min, followed by 40 cycles of denaturation at 95 °C for 15 s and annealing/extension at 60 °C for 30–40 s (with fluorescence acquisition), concluding with a melt curve analysis. RT-qPCR experiments were performed on three independent biological replicates, and statistical analysis was conducted using one-way ANOVA.

### 2.6 Western blot

For the protein expression quantification Western blot analysis was performed. Cells lysates were prepared by resuspending cell pellet into 100 µL lysis buffer made of RIPA buffer added with 1:100 protease inhibitor, 1:1000 PMSF (Phenylmethanesulfonyl fluoride, Sigma) and 1:10 Phosphatase Inhibitor (Thermo Fisher Scientific). Lysis was performed by keeping in ice for 20 min and vortexing suspensions every five minutes for a total of four times. Lysate was then clarified by 15′000 x g centrifugation for 15 min, at 4 °C and the supernatant containing the proteins were kept in ice until quantification. Lysate protein quantification was made through Lowry Protein quantification protocol with a standard curve from 0 to 1,5 μg/μL protein concentration (BioRad). Samples were prepared by diluting 15 µg of proteins into a final volume of 10.4 µL with milliQ water and added with 1,6 µL DTT (1,4-Dithiothreitol, Merck) and 4 µL Laemmli Sample Buffer (Biorad). Pre-casted gels (Thermo Fisher) were used, 16 µL of samples per well were loaded and a protein molecular weight marker (Biorad) was used. Runs were performed in a Thermo Fisher at 70 V until completion. After completion, transfer on nitrocellulose membrane with Mini Trans-Blot Electrophoretic Transfer Cell (Biorad). The membrane was then stained with Ponceau Red (Merck) and washed with TBS-Tween made with TBS added with 0,1% of Tween-20 (Merck) and cuts were made based on interest protein molecular weight. Blocking was made by membrane incubation with 5% Blotto, non-fat dry milk in TBS-Tween for 1 h at RT. Membranes were washed again with TBs-Tween and incubated with primary antibodies following the producer’s given information about dilution and diluent (antibodies information are listed in Table 2). Membranes were developed by using Clarity Western ECL Substrate (BioRad, cat no.1705061) and imaged with a Fusion FX7 imaging system (Vilber Lourmat, Torcy, France). Experiments were conducted in three biological replicates, band quantification analysis were performed through Fiji distribution of ImageJ software (National Institutes of Health, United States), and statistical analysis was conducted using one-way ANOVA.

**TABLE 2:** Antibodies for Western blot analysis.

Target	Dilution	Diluent	Code	Company
PPARγ	1:1,000	5% BSA in 1x TBST	2435	Cell signalling
GAPDH	1:1,000	5% BSA in 1x TBST	2118	Cell signalling

### 2.7 Transmission electron microscopy

After 15 days of culture, the three-dimensional cultures were collected, washed with PBS, and fixed in 2.5% glutaraldehyde (pH 7.4 in PBS) for 1 h at room temperature. Following fixation, the cultures were incubated in a 2% osmium tetroxide solution for 1 h at room temperature. The samples were then dehydrated through a graded acetone series (50%, 75%, 90%, and 100%) for 15 min per step. After dehydration, the samples were sequentially infiltrated with increasing concentrations of acetone-epoxy resin mixtures until reaching 100% epoxy resin. The resin was polymerized by heat curing at 60 °C for 24 h. The samples were sectioned using an ultramicrotome into semi-thin (1 µm) and ultra-thin (70 nm) sections. Ultra-thin sections were mounted on grids, contrasted with 2% uranyl acetate and lead citrate, and imaged using a *Talos* transmission electron microscope (Thermo Fisher Scientific).

### 2.8 Confocal microscopy

Organoids were harvested after 15 days in culture, washed with phosphate-buffered saline (PBS), and fixed in 4% paraformaldehyde (PFA, pH 7.4) for 30 min at room temperature. Following unreacted aldehyde quenching with 0.1 M glycine in PBS for 10 min, samples were permeabilized using 0.1% Triton X-100 in PBS for 10 min. Non-specific binding sites were blocked with 1% BSA in PBS for 1 h. Organoids were incubated with a primary anti-E-cadherin antibody (1:1,600, Cell Signaling) overnight at 4 °C. After washing, samples were incubated for 1 h at room temperature with an Alexa Fluor 488-conjugated goat anti-rabbit secondary antibody (1:200, Thermo Fisher Scientific). The F-actin cytoskeleton and nuclei were counterstained with Alexa Fluor 555 Phalloidin (1:400) and DAPI (1:5,000), respectively. All washing steps between incubations were performed twice with PBS. Images were acquired using a *Fluoview FV3000 laser scanning confocal microscope* (Olympus) equipped with a ×30 silicone oil immersion objective. Antibody and stain information are detailed in Table 3.

**TABLE 3:** Antibodies and stains for confocal microscopy.

Name	Target	Dilution	Diluent	Code	Company
DAPI	Nuclei	1:5,000	PBS	62248	Thermo Fisher scientific
Alexa fluor 555 phalloidin	F-actin	1:400	PBS	A34055	Thermo Fisher scientific
E-cadherin rab mAb	E-cadherin	1:1,600	1% BSA (PBS)	3195S	Cell signalling
Goat anti-rabbit IgG alexa fluor 488	Rabbit IgG	1:200	1% BSA (PBS)	A-11008	Thermo Fisher scientific

### 2.9 Statistical analysis

All experiments, were needed, were performed in at least three independent biological replicates (n ≥ 3). Data are presented as mean ± standard deviation (SD). Statistical significance was determined using one-way analysis of variance (ANOVA). Statistical analyses were performed using GraphPad Prism. A p-value of <0.05 was considered statistically significant. Exact p-values, sample sizes (n), and specific statistical tests are detailed in the respective Figure legends.

## 3 Results

### 3.1 Monolayer condition

**FIGURE 1:**
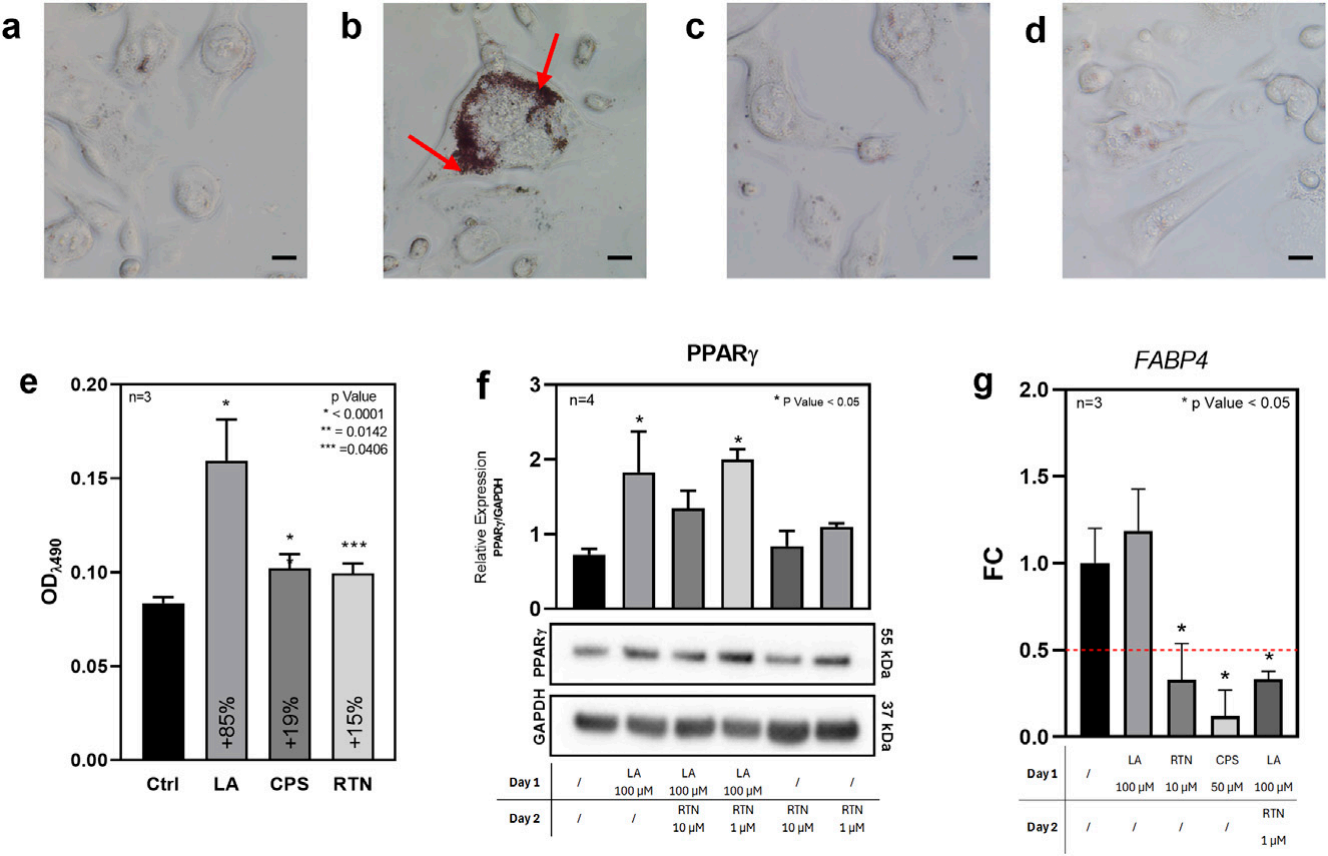
Biological characterization of SZ95 sebocytes lipid production. **(a–d)** Bright Field imaging of SZ95 sebocytes stained with Oil Red O: **(a)** Not treated cells used as control, **(b)** Linoleic Acid (LA) **(c)** Retinol (RTN) and **(d)** Capsaicin (CPS); cells were treated for 24 h before the staining procedure. Scale-bar 10 µm. **(e)** Lipids quantification through Oil Red O staining and elution. Column graphs represent SZ95 Sebocytes after 24 h of treatment with LA, CPS and RTN. Data are expressed as Optical density at 490 nm of eluted residual staining. Untreated cells used as control (Ctrl). **(f)** Western blot analysis of PPARγ expression. The bar graph shows the quantification of PPARγ protein levels in SZ95 sebocytes treated with LA, or 10 μM and 1 µM RTN. Untreated cells served as a control (CTRL). Data are expressed as the PPARγ/GAPDH optical density ratio. **(g)** Analysis of FABP4 gene expression by qPCR. The bar graph shows FABP4 mRNA levels in SZ95 sebocytes treated for 24 h with LA, CPS, or RTN. Untreated cells served as CTRL. Data are expressed as fold change relative to the control, after normalization to B2M expression.

Sebocytes cultured in monolayer (Figure 1a) were exposed to linoleic acid (LA) (Figure 1b) to induce a lipidogenic phenotype and subsequently treated with pharmacological inhibitors RTN (Figure 1c) and CPS (Figure 1d). LA stimulation induced a marked accumulation of intracellular lipid droplets (Figure 1b, red arrows). Quantitative Oil Red O analysis (Figure 1e) demonstrated a significant increase in neutral lipid content in LA-treated cells (+85% compared to vehicle control, p < 0.0001). Sequential treatment with RTN and CPS significantly reduced lipid accumulation of 66% and 70% relative to LA alone (p = 0.0142 and p = 0.0406), indicating effective modulation of lipid storage.

At the molecular level, Western blot analysis revealed that LA treatment significantly increased PPARγ protein levels compared to control conditions (Figure 1f, p < 0.05). RTN treatment reversed this effect in a dose-dependent manner, with the 10 µM concentration inducing a marked reduction in PPARγ expression compared to the LA group. Consistently, gene expression analysis demonstrated that FABP4 mRNA levels (Figure 1g) were upregulated in response to LA stimulation (Fold Change: 1.18, p < 0.05). Treatment with RTN and CPS resulted in a significant downregulation of FABP4 expression, paralleling the morphological data.

### 3.2 3D sebaceous gland reconstruction

**FIGURE 2:**
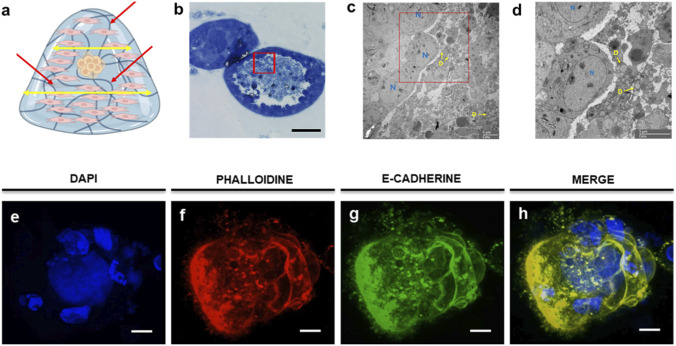
Design and morphological characterization of SZ95 sebocyte organoids. **(a)** SZ95 sebocytes and human dermal fibroblasts organoid design. **(b)** Brightfield micrograph of a semithin organoid section, scalebar = 60 µm. **(c,d)** TEM micrographs of an SZ95 organoid at different magnifications. **(e-h)** Confocal fluorescence images of an SZ95 organoid, scale bar = 5 µm.

To model the structural organization of the sebaceous gland within a dermal-like niche, we engineered a 3D co-culture organoid comprising SZ95 sebocytes and hDFs (Figure 2a). The organoids displayed a compartmentalized architecture characterized by a compact, spheroid inner region and a peripheral layer of elongated spindle-shaped cells (Figure 2b). Morphological evaluation of the central core revealed increased cytoplasmic granularity and refractive inclusions. Transmission electron microscopy (Figures 2c,d) confirmed the presence of cytoplasmic lipid vacuoles and membrane-bound vesicular structures within the inner cells, while the peripheral compartment consisted of cells with extended cytoskeletal networks (Figure 2c, yellow arrows). Cell–cell interfaces were observed at the boundary between the two populations (Figure 2d, red square). Immunofluorescence analysis confirmed the spatial organization of the co-culture (Figures 2e-h). E-cadherin staining (Figure 2f, 2red) was strongly localized to the membranes of the inner sebocyte compartment, confirming epithelial identity. Phalloidin staining (Figure 2g, green) highlighted F-actin structures predominantly within the peripheral fibroblast-like cells.

**FIGURE 3:**
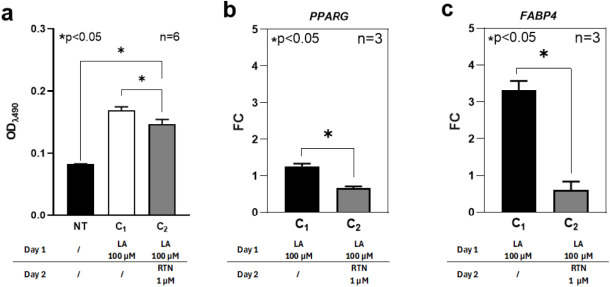
Lipid quantification and lipid metabolism-related gene expression in 3D sebaceous gland organoids (SGO). **(a)** Quantification of intracellular lipid accumulation via Oil Red O staining extraction (absorbance at 490 nm) **(b,c)** Relative mRNA expression levels (Fold Change, FC) of PPARG **(b)** and FABP4 **(c)**. To evaluate the modulatory effect, organoids were subjected to a sequential treatment scheme: untreated controls (NT); an initial 24-h stimulation with 100 µM linoleic acid (LA) on Day 1 followed by vehicle control on Day 2 (C1); and LA stimulation on Day 1 followed by an additional 24-h exposure to 1 µM retinol (RTN) on Day 2 (C2). Data is presented as mean ± SD. The number of independent biological replicates is n = 6 for the lipid quantification assay and n = 3 for the gene expression analysis. Statistical significance was determined using one-way ANOVA (*p < 0.05).

To evaluate sebogenic competence in 3D, organoids were subjected to lipidogenic assays (Figure 3). LA stimulation significantly increased intracellular lipid accumulation within the central compartment, as quantified by Oil Red O extraction (Figure 3a, p < 0.05). Pharmacological inhibition with RTN significantly attenuated this lipid deposition. Correspondingly, LA stimulation induced the upregulation of PPARγ and FABP4 mRNA transcripts, which were subsequently downregulated upon RTN treatment (Figures 3b,c, p < 0.05).

**FIGURE 4:**
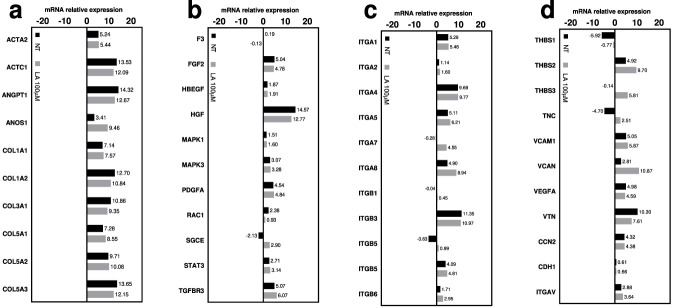
Transcriptional profiling of extracellular matrix and adhesion molecules in 3D sebaceous gland organoids. Relative mRNA expression levels of SZ95 and hDF co-culture organoids were evaluated following 24 h of treatment with 100 µM linoleic acid (LA) compared to untreated controls (NT). Data are presented as relative mRNA expression levels. **(a)** Structural matrix and cytoskeletal components. **(b)** Signaling molecules and growth factors. **(c)** Integrin subunits, showing targeted enhancement of specific cell-matrix adhesion receptors. **(d)** Matricellular proteins and structural glycoproteins, highlighting substantial upregulations of Versican (VCAN) and Thrombospondins (THBS2, THBS3).

To evaluate microenvironmental remodeling upon sebogenic activation, we analyzed the expression of extracellular matrix (ECM) components, adhesion molecules, and associated signaling factors in 3D organoids following 100 µM LA stimulation (Figure 4). The array revealed coordinated transcriptional shifts across multiple functional clusters. Among matrix and cytoskeletal genes (Figure 4a), fibrillar collagens (e.g., COL1A1, COL1A2, COL3A1) maintained robust basal expression with minor modulations, whereas ANOS1 exhibited a marked upregulation, increasing its relative expression from 3.41 in untreated (NT) controls to 9.46 in LA-treated organoids. Profiling of signaling molecules and growth factors (Figure 4b) showed a notable positive shift for SGCE (from −2.13–2.90) and a downregulation of RAC1 (from 2.38 to 0.93), while HGF expression remained high but slightly decreased (14.57–12.77).

The integrin expression profile (Figure 4c) highlighted a targeted enhancement of specific cell-matrix adhesion receptors upon LA stimulation. Specifically, we observed strong inductions for ITGA7 (from −0.28–4.55) and ITGA8 (from 4.90 to 8.94), while other subunits such as ITGB3 (11.35–10.97) and ITGB5 (4.09–4.81) maintained stable positive expression levels. The most pronounced transcriptional alterations were observed among matricellular proteins and structural glycoproteins (Figure 4d). LA stimulation induced substantial upregulations of Versican (VCAN, from 2.81 to 10.87) and members of the Thrombospondin family, notably THBS2 (from 4.92 to 9.70) and THBS3 (from −0.14–5.81). Furthermore, Tenascin-C (TNC) expression transitioned from negative to positive values (from −4.70 to 2.51), concurrent with a reduction in Vitronectin (VTN, from 10.30 to 7.61).

**FIGURE 5:**
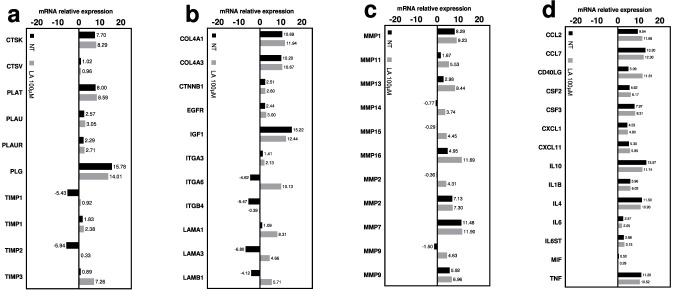
Transcriptional profiling of proteases, basement membrane components, and cytokines in 3D sebaceous gland organoids. Relative mRNA expression levels of SZ95 and hDF co-culture organoids were evaluated following 24 h of treatment with 100 µM linoleic acid (LA) compared to untreated controls (NT). Data are presented as relative mRNA expression levels. **(a)** The plasminogen activation system and tissue inhibitors of metalloproteinases (TIMPs), with notable upregulation of TIMP2 and TIMP3. **(b)** Basement membrane constituents and epithelial adhesion markers, showing marked induction of specific laminins (LAMA3, LAMB1) and ITGA6. **(c)** Matrix metalloproteinases (MMPs), indicating active, localized matrix remodeling. **(d)** Inflammatory and immune-modulatory cytokines.

To further characterize the functional state of the 3D organoids, we expanded our transcriptomic analysis to include matrix metalloproteinases (MMPs), tissue inhibitors of metalloproteinases (TIMPs), basement membrane components, and cytokines (Figure 5). Analysis of proteases and their inhibitors (Figure 5a) revealed that while the plasminogen activation system (e.g., PLAT, PLAU, PLG) remained highly expressed with minimal variations, TIMPs underwent significant upregulation. Notably, TIMP2 expression shifted from −5.94 to 0.33, and TIMP3 sharply increased from 0.89 to 7.26 upon LA stimulation.

The basement membrane and epithelial adhesion cluster (Figure 5b) displayed some of the most striking transcriptional transitions. The alpha-6 integrin subunit (ITGA6) shifted dramatically from −4.52 in untreated controls to 10.13 in LA-stimulated organoids. Several laminin chains transitioned from negative values to robust positive expression, specifically LAMA3 (from −5.50 to 4.95) and LAMB1 (from −4.12–5.71), while LAMA1 increased from 1.05 to 6.31. MMP profiling (Figure 5c) confirmed the widespread activation of extracellular proteases. MMP13 increased from 2.96 to 8.44, and MMP11 rose from 1.67 to 5.53.

Furthermore, several matrix metalloproteinases transitioned from negative to positive relative expression following lipidogenic stimulation, notably MMP14 (from −0.77–3.74) and MMP9 (from −1.50–4.63). The assessment of inflammatory and immune-modulatory genes (Figure 5d) showed targeted alterations. While most interleukins (e.g., IL10, IL1B, IL4) and TNF maintained stable high expression with slight decreases, specific factors were upregulated: CCL2 increased from 7.54 to 11.89, and CD40LG expression rose from 5.09 to 11.51.

## 4 Discussion

**FIGURE 6:**
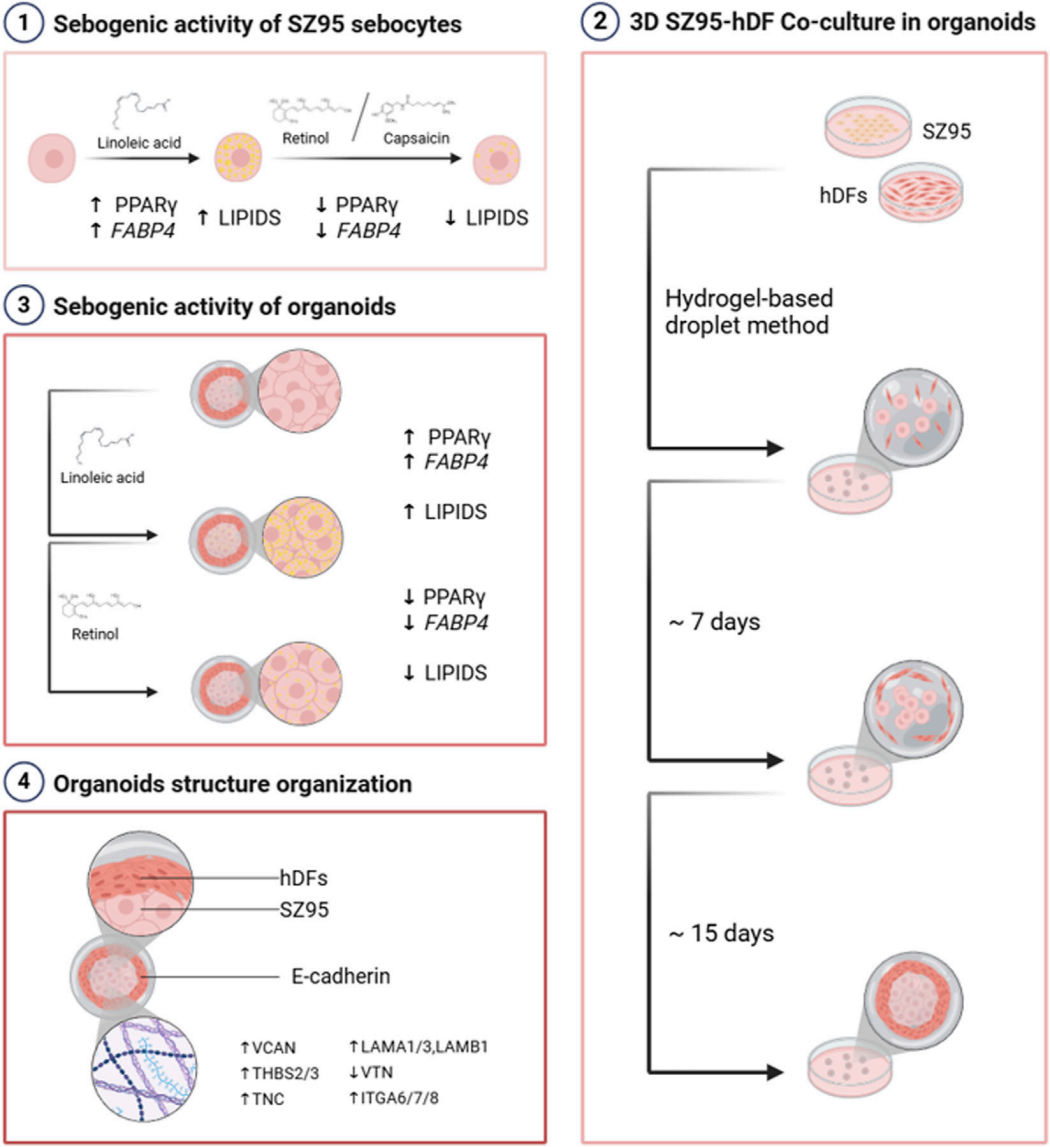
Production and characterization of 3D Sebaceous Gland Organoids. **(1)** Lipid modulation and gene expression (PPARγ, FABP4) in 2D SZ95 sebocyte cultures using sebogenic (linoleic acid) and anti-sebogenic (retinol, capsaicin) compounds. **(2)** Self-assembly of SZ95 cells and human dermal fibroblasts (hDFs) into 3D organoids. **(3)** Functional validation showing the mature 3D organoids’ responsiveness to sebogenic modulators. **(4)** Spatial and molecular architecture of day-15 organoids, featuring an hDF outer shell, an E-cadherin-expressing SZ95 inner core, and a specialized extracellular matrix (ECM) network.

In this study, we developed a matrix-supported 3D co-culture model that mimics the spatial compartmentalization of the human sebaceous gland within a stromal-like microenvironment, as summarize in Figure 6. While traditional monolayer cultures of immortalized sebocytes have provided valuable insights into lipid metabolism, they lack the mechanical and paracrine cues necessary to model glandular architecture. Our 3D model integrates a sebocyte-rich core with an external hDF-populated dermal equivalent, allowing for the evaluation of lipidogenic responses in a more structurally complex context (Figure 6, box 2).

Our data demonstrated that linoleic acid (LA) stimulation robustly activates the sebogenic program in 3D, evidenced by intracellular lipid deposition and the upregulation of key metabolic regulators, PPARγ and FABP4. Beyond the induction of lipid biosynthetic pathways, our transcriptional analysis revealed that sebogenic activation in 3D elicits a profound and coordinated microenvironmental remodeling response (Figure 6, box 3).

The transcriptomic profile of LA-stimulated organoids shows a dynamic reorganization of the extracellular space, driven by the marked induction of key matricellular proteins and adhesion molecules. The substantial upregulation of Versican (VCAN) and Tenascin-C (TNC) - macromolecules typically associated with tissue remodeling, viscoelasticity, and wound healing-suggests an active adaptation of the stromal compartment. Expanding lipid-laden sebocytes generate mechanical and spatial demands; the shift toward a TNC/VCAN-enriched provisional matrix, alongside a reduction in rigid components like Vitronectin (VTN), likely increases matrix pliability, accommodating glandular hypertrophy while preventing stromal compression.

This is further supported by the significant induction of Thrombospondins (THBS2 and THBS3), matricellular proteins that modulate cell-matrix interactions, balance local angiogenic cues, and manage cellular stress within expanding tissues. Concurrently, the robust upregulation of specific integrin subunits (such as ITGA7 and ITGA8) and ANOS1 points toward a compensatory reinforcement of epithelial-stromal anchorage. As the glandular core undergoes lipid-driven expansion, the concurrent enhancement of these adhesion receptors ensures that epithelial integrity and mechano-transduction pathways are maintained. Rather than representing a disorganized structural breakdown, these transcriptional shifts indicate that sebogenesis is functionally coupled with a highly controlled, regenerative-like adaptation of the dermal niche, ensuring architectural coherence during active lipid accumulation.

Our gene expression data further elucidate the sophisticated cross-talk between the epithelial sebaceous core and the dermal-like stroma. The robust, concurrent upregulation of various matrix metalloproteinases (MMPs), including MMP9, MMP13, and MMP14, confirms an active, localized degradation of the provisional matrix to accommodate the volume expansion of lipid-accumulating sebocytes. This proteolytic activity, however, is not uncontrolled; the simultaneous induction of specific tissue inhibitors of metalloproteinases, particularly TIMP2 and TIMP3, indicates a tightly regulated remodeling process. This MMP/TIMP balance prevents excessive stromal degradation, ensuring that tissue architecture is preserved during glandular hypertrophy.

Another key physiological finding is the strong upregulation of basement membrane constituents following LA stimulation. The striking induction of Laminins (LAMA3, LAMB1) alongside the alpha-6 integrin subunit (ITGA6) - a classic hemidesmosome marker - strongly suggests that the metabolically active sebocytes are attempting to establish a mature basement membrane interface. *In vivo*, the pilosebaceous unit is sharply demarcated from the surrounding dermis by a specialized basement membrane that regulates bidirectional signaling and structural polarity. The transition of these markers from negative to highly positive expression indicates that our 3D model partially mimics this *in vivo* compartmentalization mechanism, actively reinforcing the epithelial-mesenchymal boundary in response to sebogenic activation.

Finally, the selective modulation of cytokines, notably the upregulation of CCL2 and CD40LG, amidst a stable background of baseline interleukins, suggests that the organoids preserve immune-modulatory competence. Since native sebaceous glands actively participate in cutaneous innate immunity and neuroendocrine signaling, the maintenance of this cytokine profile confirms the potential of this 3D platform to model not only sebogenesis but also the inflammatory cross-talk central to sebaceous gland disorders like acne vulgaris.

While the model shows promise, a few limitations remain before it can be fully translated to regulatory toxicology or advanced preclinical screening. Because our model relies on the immortalized SZ95 cell line, a standard surrogate, it may not completely capture the holocrine secretory profile of primary sebocytes. Moving forward, incorporating primary cells or iPSC-derived progenitors would improve physiological relevance.

Additionally, because Oil Red O non-specifically stains all neutral lipids, it cannot definitively differentiate generic lipid storage from sebum-specific production. Although our PPARγ and FABP4 data support a sebogenic shift, subsequent studies will require comprehensive lipidomic profiling or gas chromatography-mass spectrometry (GC-MS) to confirm the synthesis and secretion of specific markers like squalene. We also plan to integrate a broader panel of terminal differentiation markers (e.g., KRT7, MUC1, or specific lipid synthesis enzymes) to better validate the holocrine differentiation trajectory within the organoid. Addressing these gaps will further refine the model, offering a robust, animal-free platform for dermatological and cosmetic research.

## Data Availability

The raw data supporting the conclusions of this article will be made available by the authors, without undue reservation.
